# Portuguese Validation of the Unified Theory of Acceptance and Use of Technology Scale (UTAUT) to a COVID-19 Mobile Application: A Pilot Study

**DOI:** 10.3390/healthcare11131916

**Published:** 2023-07-03

**Authors:** Ivandra Araújo, Ana Grilo, Carina Silva

**Affiliations:** 1 ESTeSL—Escola Superior de Tecnologia da Saúde, Instituto Politécnico de Lisboa, Av. D. João II, Lote 4.69.01., 1990-096 Lisboa, Portugal; ivandra.10@gmail.com; 2 H&TRC—Health & Technology Research Center, ESTeSL—Escola Superior de Tecnologia da Saúde, Instituto Politécnico de Lisboa, Av. D. João II, Lote 4.69.01., 1990-096 Lisboa, Portugal; ana.grilo@estesl.ipl.pt; 3 CICPSI, Faculdade de Psicologia, Universidade de Lisboa, Alameda da Universidade, 1649-013 Lisboa, Portugal; 4 Centro de Estatística e Aplicações, Faculdade de Ciências da Universidade de Lisboa, Bloco C6-Piso 4, 1749-016 Lisboa, Portugal

**Keywords:** mobile application, UTAUT scale, COVID-19, scale validation, digital health

## Abstract

The use of technology has proven to be a powerful tool in the fight against COVID-19 and its variants of concern (Gamma, Beta, Alpha, Delta, and Omicron). The urgency of responding to this worldwide pandemic has accelerated the development of monitoring systems and contact tracing applications. Without technology’s contribution, the adverse effects on humanity economically, socially, and psychologically would be even more devastating. This study aimed to translate, adapt, and validate the Unified Theory of Acceptance and Use of Technology (UTAUT) model in a Portuguese university population, to evaluate adherence to a mobile application for tracking COVID-19. An observational cross-sectional study was developed using a sample with 1081 participants (71% female, 59.2% with ages between 16 and 24, and 78.2% of the respondent’s university students). The Portuguese version model showed a good reliability (Cronbach’s α = 0.82) and an acceptable overall adjustment to the sample under study (χ^2^/df = 3.732, CFI = 0.955, TLI = 0.944, RMSEA = 0.05, SRMR = 0.06); however, this model could be improved, as we proved. Since this is a pilot study, more studies are needed. The results indicated that the P-UTAUT can be improved for evaluating adherence to a COVID-19 mobile application.

## 1. Introduction

Public healthcare expenditure has increased in all European Union (EU) Member States [1]. The scarcity of human resources and the rise of chronic diseases, especially among older age groups, have contributed to this expense increase. The COVID-19 pandemic has further accelerated this upward trend. Therefore, new technologies emerge as a powerful tool to ensure the sustainability of medical services without compromising quality. Cultural, economic, technical, and political barriers, as well as the level of digital literacy and disparities in internet access, are determining factors for the success of mobile technologies. Despite the positive investments in empowering and increasing digital literacy among the Portuguese population, much remains to be accomplished.

Digital medicine has become indispensable in the fight against the COVID-19 pandemic by enabling remote monitoring of patients, surveillance, and control through the collection of relevant data for timely clinical decision-making, thereby saving lives. Technology has also enabled new solutions, such as telecommuting, online classes, and teleconsultations. Simultaneously, several countries have developed digital tracing mobile applications that calculate the individual risk of exposure to the SARS-CoV-2 virus and identify possible contagion sources. Among the various tools created by different countries are contact tracing applications that assist Public Health Authorities in rapidly tracing at-risk contacts. However, the acceptance of these apps among the public significantly impacts their effectiveness. It is, therefore, essential to find measures to evaluate the population’s adherence to these devices. In this field, Walrave et al. (2020) adapted a scale based on the Unified Theory of Acceptance and Use of Technology model (UTAUT) for the “Ready or Not for Contact Tracing” study to explain the factors that influence the adoption of contact tracing applications [2]. 

Portugal has created the STAYAWAY COVID smartphone application to contain the COVID-19 virus from spreading. It is free to use, voluntary, and anonymous, and it was introduced in full on 1 September 2020. The notification process begins with the installation of the STAYAWAY COVID app. A unique code, called a TEK (Temporary Exposure Key), is created daily. Based on this key, alphanumeric identifiers known as RPIs (Rolling Proximity Identifiers) are generated every 10 min. The RPI is broadcasted and captured by nearby mobile devices with the app installed using the Bluetooth Low Energy (BLE) communication protocol. If a user is diagnosed with the SARS-CoV-2 virus, the doctor accesses the Diagnosis Legitimization Server (DLS) to obtain the legitimation code (CL), which is delivered to the user in paper format via message or email. With the CL, the user can voluntarily enter it into the application, triggering the automatic transmission of TEK codes to the Diagnosis Publication Server (DPS). In turn, the DPS will compare the infected user’s RPI with the RPIs of the mobile devices with which they had contact and calculate the risk of exposure. The criteria for this calculation are the period, proximity, and time, which means all contacts within the last 14 days, at a distance of less than 2 m, and lasting for more than 15 min. If the risk is confirmed, the application alerts the user(s) about the necessary procedures. Having Bluetooth active during the exposure is mandatory to receive this notification.

In terms of using new technologies, Portugal is in a good position. It has been a world reference in several fields regarding Information and Communications Technology (ICT) development [3]. For example, the “Multibanco” network (automated teller machine—ATM) is one of the most sophisticated banking networks in the world, “Via Verde” was the first closed system of automatic highway tolls in the world, and Pre-Paid Mobile Phones became the foundation for the mobile revolution we live in today [3]. Within the European countries, Portugal has one of the highest rates of FTTH (Fiber to the home) penetration, and according to official data from the National Statistics Institute (INE), in 2020, 84.5% of households had an internet connection at home [4].

Portugal is not only a market open to innovation, but also the population’s adherence to health technologies is remarkable. For example, in terms of vaccination, according to Our World in Data [5], on 24 December 2021, Portugal was the 14th European Union country that administered the most vaccines per 100 inhabitants, with a rate of 89.21% of the population fully vaccinated. Therefore, it becomes even more relevant to validate a model that allows evaluating the technology acceptance not only for contact tracing applications, but also for all m-health tools created.

Several models attempt to study the behavioral dimensions that explain technology adoption. However, one of the most extensive explanations of technology adoption available can be obtained by UTAUT [6] and has been widely used to explain technology adoption in different contexts and groups, namely, in healthcare settings. Further, it has 2 other advantages: (1) UTAUT can explain up to 70% of the variation in the intention to use a given technology [7,8], and (2) it considers both technological aspects and social factors [9]. Venkatesh et al. (2003) [7] created the Unified Theory of Acceptance and Use of Technology (UTAUT) model in 2003, based on eight other models of individual acceptance, namely, the Theory of Reasoned Action (TRA), Theory of Planned Behavior (TPB), Motivational Model (MM), Technology Acceptance Model (TAM and TAM2), Social Cognitive Theory (SCT), Model of PC Utilization (MPCU), and Innovation Diffusion Theory (IDT) [7]. 

The UTAUT model has four primary constructs explaining the intention to use a particular technology: performance expectancy, effort expectancy, social influence, and facilitating conditions [7,10]. Walrave et al. (2020) adapted the model by including three more constructs: (1) app-related privacy concerns, (2) innovativeness, and (3) COVID-19-related stress [2]. In the present study, it is used the Walrave et al. (2020) version, where the following constructs were used:

1. Performance expectancy refers to the benefits expected from using a given technology [2,7].
2. Effort expectancy directs the individual’s perception of the difficulty using a given technology [2,7].
3. Social influence refers to the individual’s perception of other people’s opinions about a particular technology [2,7].
4. Facilitating conditions direct all the resources needed to use a particular technology [7,10].
5. App-related privacy concerns are related to the risk of personal data breaches [11].
6. Innovativeness relates to individuals predisposed to buy or adopt new products [2].
7. COVID-19-related stress is defined as the individual’s perception of adopting protective measures when perceiving a health risk [7,12].

The urgency to respond to the global pandemic situation has led to an acceleration in the development of monitoring technologies and decision support systems. More than a year after the launch of the first app, the need to evaluate the adherence and devise strategies to encourage increased adoption has emerged.

The present study aims to translate and validate the UTAUT model developed by Walrave et al. (2020) [2] to evaluate the adherence of the mobile application STAYAWAY COVID in a Portuguese university population.

## 2. Materials and Methods

### 2.1. Participants and Data Collection

An observational, cross-sectional study was conducted using a non-probability and voluntary response sampling method at Portuguese Polytechnic Institutes. Approval was requested to apply the questionnaire to all the 20 Polytechnic Institutes (private and public) in Portugal, where 4 were accepted. The inclusion criteria of the participants were to express their willingness to participate in the study through informed consent, the ability to read and write in Portuguese, and possessing an institutional email account. The data were collected anonymously and voluntarily using an online form and disseminated via institutional email to all participants who met the inclusion criteria. The questionnaire access link was sent to the Polytechnic Institutes on 1 March 2021, for subsequent dissemination to their respective academic communities. The data collection process was extended until 1 April 2021. It was obtained a total of 2018 participants.

### 2.2. Ethical Considerations

The study was conducted following the Declaration of Helsinki and approved by the Ethics Committee of Escola Superior de Tecnologia da Saúde de Lisboa (protocol code CE-ESTeSL-No. 69-2020 approved on 23 November 2020). Authorization was obtained from the original author (Michel Walrave) for the translation and use of the questionnaire UTAUT. All participants were informed of the study’s objectives, authors, purpose, and data collection type. Participation in the study was voluntary and subject to acceptance of the proposed terms outlined in the informed consent. In order to reinforce the confidentiality of participant data and address the ethical issues related to data collection, none of the questions posed allow for participant identification.

### 2.3. Questionnaire

**Figure 1:**
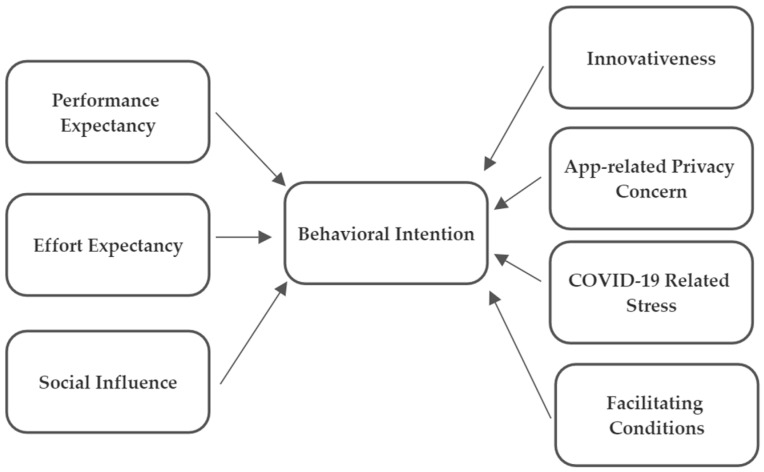
UTAUT model framework.

The final UTAUT model comprises eight constructs (behavioral intention, performance expectancy, effort expectancy, social influence, facilitating conditions, innovativeness, app-related privacy, and COVID-19-related stress), where this model intends to predict an individual’s behavioral intention and use behavior of technology (Figure 1). Each construct comprises 3 items, totaling 24 items (Table 1). The response to each item is given according to a five-point Likert scale, where one means “strongly disagree” and five means “strongly agree”, except for the App-related privacy construct, where the items are in reverse scale. Thus, answer one means “strongly agree” with this construct, and five means “strongly disagree”. Items negatively worded were reverse-scored for further analysis. The total score of the questionnaire ranges between 24 and 120 points, and the higher the score, the higher the behavioral intention.

It also asked gender, age group, educational qualification, occupation, and open questions to identify participants’ perceptions of the mobile application, for example: (1) Have you heard of the contact tracing mobile application? (2) Have you installed the contact tracing application?

### 2.4. Translation Process

**Figure 2:**
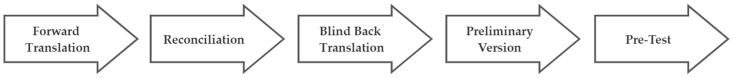
Translation process.

The translation process was developed in five steps, according to the guidelines presented by Vijver et al. (2006) [13] and by Sousa et al. (2011) [14], as shown in Figure 2 and described next.

1. Forward translation: the scale was independently translated into Portuguese European language by four native speakers of Portuguese who were fluent in English.
2. Reconciliation: the four versions were compared, and a single version was produced.
3. Blind back-translation: the previous version was subjected to a back-translation. It was explained to the translator the study’s objectives without showing the original questionnaire.
4. Preliminary version: the versions obtained in the second and third steps were compared, and a preliminary version was created.
5. Pre-test: the questionnaire obtained in the fourth step was subjected to a pre-test to identify possible semantics errors and ambiguous questions. A total of 5 individuals from the general population participated in the pre-test: 3 men and 2 women, aged between 18 and 45. Most participants indicated that some consecutive items seemed to have the same meaning. Therefore, to reduce this ambiguity, we randomly ordered the sentences. The participants also suggested eliminating the pronouns from the sentences so that reading becomes less tiring. Corrections were made, and the final questionnaire was obtained (Table 1).

### 2.5. Data Analysis

The validity of the scale was assessed through (1) reliability analysis, (2) validity analysis, and (3) goodness of fit indices. A Confirmatory Factor Analysis (CFA) was performed.

Reliability analysis: Cronbach’s alpha was calculated. According to Bolarinwa (2015) [14], an instrument is classified as consistent (reliable) when the alpha (α) is at least 0.70. However, some social science research allows a Cronbach’s alpha of 0.60, yet the results should be read cautiously and assess the context in which the index is computed [15]. In that sense, it was also obtained that the Composite Reliability that was 0.7 is the minimum acceptable.

Validity analysis: factor loadings (λ_ij_) were calculated and considered acceptable when λ_ij_ > 0.5. However, some authors defend acceptable values when λ_ij_ ≥ 0.216 [16]. Additionally, to access convergent validity, which indicates the extent to which different items that measure the same construct are correlated with each other, it was calculated the average variance extracted (AVE), and it was considered acceptable when AVE ≥ 0.5.

Goodness of fit indices: A CFA was performed, and the goodness of fit indices were obtained. The following indices were used: chi-square (χ^2^), chi-square divided by the number of degrees of freedom (χ^2^/*df*), Standardized Root-Mean-Squared Residual (SRMR), Comparative Fit Index (CFI), Tucker–Lewis Index (TLI), and Root-Mean-Square Error of Approximation (RMSEA). Statistical analysis was performed using the software IBM SPSS—version 26.0 and IBM SPSS Amos 26.0.

## 3. Results

### 3.1. Descriptive Statistics

As shown in Table 2, 1081 participants (students, teachers, and other school staff) completed the questionnaire. Of these, 71% were female, 59.2% were between 16 and 24 years old, 78.2% were students, and 41.2% had a bachelor’s degree.

### 3.2. Reliability

It was obtained a global Cronbach’s alpha of 0.82, which can be considered good, according to the classification presented by Taber et al (2018) [17]. In addition, the constructs (1) facilitating conditions, (2) innovativeness, and (3) COVID-19-related stress had a Cronbach’s alpha less than 0.60 (considered weak) [18] (Table 3).

### 3.3. Validity

Factorial validity: As reported in Table 3, all the items had values λ_ij_ ≥ 0.216. Therefore, all were included in the final questionnaire.

Convergent validity: In Table 3, the constructs (1) Effort expectancy, (2) Facilitating conditions, (3) Innovativeness, and (4) COVID-19-related stress have not demonstrated convergent validity in this sample since the AVE was less than 0.5.

Discriminant validity: This analysis was conducted using the matrix of correlations (Table 4).

The correlation between the constructs’ effort expectancy and facilitating conditions is higher than 0.85, meaning that the 2 constructs could be combined, and some items eliminated.

### 3.4. Goodness of Fit Indices

**Figure 3:**
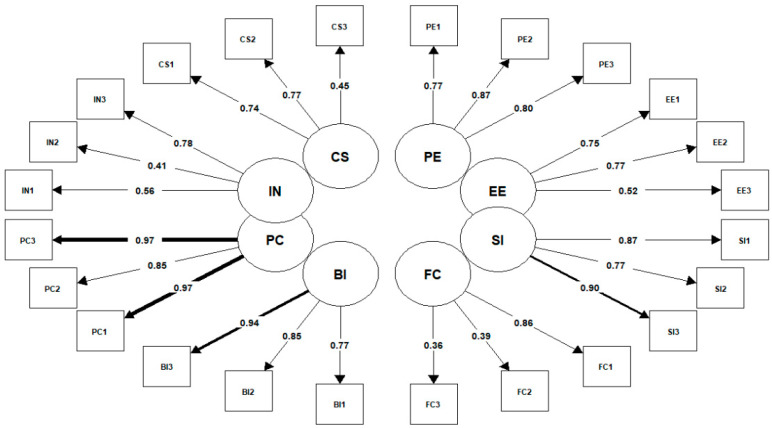
Path diagram of the confirmatory factor analysis.

Figure 3 represents the CFA-proposed model P-ATAUT as a path diagram. Arrows represent the relationship between factors or dimensions (in circles) and items (in squares). The coefficients on the arrows are the factors loadings (or eigenvalues) that show the strength of those relationships. All coefficients were higher than 0.2 and ranged between 0.341 and 0.967.

As shown in Table 5, the initial model indicates an acceptable model fit (χ^2^/df = 3.732, *p* < 0.001; RMSEA = 0.050, IC 90% [0.047–0.054]). Modifications were performed on the initial model, where the following constructs were excluded: (1) Effort expectancy, (2) Facilitating conditions, and (3) Innovativeness. These modifications caused an improvement in all the goodness of fit indices considered: χ^2^/df = 2.6, *p* < 0.001; RMSEA = 0.047, IC 90% [0.041–0.052] (Table 5).

## 4. Discussion

The objectives of this study were (1) to translate the UTAUT scale adapted by Walrave et al. (2020) [2] into the European Portuguese language and (2) to validate the UTAUT scale in the same language. The analysis results indicated that the P-UTAUT scale exhibited good reliability and validity in our sample, where the majority of the goodness of fit indices showed a good adjustment of the P-UTAUT model. Only the χ^2^/*df* showed poor fit; however, it is important to note that there is no universally agreed-upon cutoff for this fit index [19,20,21,22], and it should not be analyzed by itself. In total, 3 constructs, namely, (1) Facilitating conditions, (2) Innovativeness, and (3) COVID-19-related stress, demonstrated a weak Cronbach’s alpha value of less than 0.70. In contrast, the study conducted by Walrave et al. (2020) [2] obtained a Cronbach’s alpha of 0.89 for facilitating conditions and 0.76 for innovativeness, which were considered good and reasonable, respectively. Furthermore, in the studies conducted by Hoque et al. (2017) [23] and Apolinário-Hagen et al. (2018) [24], facilitating conditions also demonstrated a good Cronbach’s alpha of 0.85 or higher. The difference in the factorial structure between our study and previous studies could be partially attributed to the characteristics of our sample. For example, Walrave et al. (2020) [2] did not focus on an academic community and employed a stratified sampling procedure to achieve a more diverse sample. Our sample consisted mostly of females (71%) with an average age of 28 years old, while Walrave et al.’s [2] sample had a balanced distribution of males and females with an average age of 41.58. Regarding the goodness of fit indices, the initial model had fit indices of χ^2^/df = 3.732, CFI = 0.955, and TLI = 0.944, which are similar to those obtained in the study developed by Walrave et al. (2020) [2] (χ^2^/df = 3.63, CFI = 0.974, and TLI = 0.963). However, Kukuk (2020) [6] obtained better values for the following indices: χ^2^/df = 1.737 and CFI = 0.922. Paganin et al. (2022) [25] conducted a study to investigate the acceptance of mobile apps for mental health in Italy and Germany in university students, where they confirm the invariance of the scales. On the other hand, after conducting some modifications to the initial model, such as excluding the constructs (1) effort expectancy, (2) facilitating conditions, and (3) innovativeness, all the indices have improved significantly. Although more evidence is needed to understand why this difference occurred, it could be explained by the study sample (academic community), who use technologies frequently and efficiently, making these constructs irrelevant here. Many participants reported that they would have no difficulty learning to use the application. For example, 79.1% of the participants answered “agree” or “strongly agree” to the statement “Learning how to use the COVID-19 app will be easy for me” (EE1), 94.7% answered “agree” or “strongly agree” to the statement “I have the necessary resources to use the COVID-19 app” (FC2), and 77.8% answered “agree” or “strongly agree” to the statement “I can usually figure out how to use new high-tech products and services without help from others” (IN3). Consistent with previous research [2,6,7], the fundamental constructs for Behavioral Intention were identified as performance expectancy and social influence. Thus, a positive perception of the performance of a given mobile application and other individuals’ opinions about their experience with a particular application may increase the likelihood of m-health adoption. Furthermore, in our sample, composed mainly of young people aged between 16 and 34 (73.5%), COVID-19-related stress did not demonstrate relevance. This finding is consistent with the study conducted by Rosi et al. (2021) [26], where the perception of risk severity increased with age from young to older adult groups. Similarly, in a study conducted after the first lockdown period in Switzerland, Franzen & Wöhner (2021) [27] noticed that young adults perceived themselves as at low personal risk of COVID-19. Therefore, considering that the main limitation of this study is related to sample homogeneity, although the psychometric analysis of the P-UTAUT questionnaire points to the removal of three constructs, further studies with representative samples of the Portuguese population should be conducted.

## 5. Conclusions

Technologies, such as COVID-19 mobile applications, provide a cost-effective way to reach large numbers of people and reduce pressure on hospitals; it is critical to analyze the factors that influence the adoption of health technologies. Only by doing so is it possible to define more efficient measures. Performance expectation emerges as the construct most associated with the intention to use and the intention to continue using STAYAWAY COVID in the academic community. In fact, participants’ main criticisms of the application are related to its performance, specifically in the notification process and code generation. People indicated that (1) they did not receive codes even after multiple requests and (2) they were not notified by the application even after contact with infected family/friends. The perception of the application’s low performance led to its uninstallation by some individuals. If people do not receive the codes to notify, which is one of the main functions of the application, it loses its “strength” and usefulness. As this construct is crucial for the usage of STAYAWAY COVID, it is important to create solutions that promote improvement in its performance. It is suggested that obtaining the code does not depend on healthcare professionals, who are overwhelmed with their daily routines, but rather be a more expedited process, for example, utilizing the PCR test code. Social influence also proved to be an important construct for the intention to use the STAYAWAY COVID application. Approximately 62% of individuals who do not use the application revealed that those closest to them do not recommend its use. STAYAWAY COVID is a tool with great potential for combating a pandemic. However, the reduced perception of its functionality and performance has hindered an increase in its adoption rate.

Questionnaire validation is a crucial aspect of health technology assessment, particularly for technologies used by the general public. Regarding COVID-19 mobile applications, questionnaire validation ensures that the app provides accurate and reliable information to users and healthcare providers. It allows decision-makers to analyze the factors that may influence the adoption of m-health and define strategies to increase usage and create effective advertising. Nowadays, COVID-19 is not an issue; however, the potential for diseases to spread is increasing, the risk of outbreaks escalating into epidemics or pandemics is considerable, and mobile technologies are very useful tools to minimize the spread. Our results support that the modified P-UTAUT questionnaire for this purpose in Portugal is a good tool to achieve the adherence to a mobile app; however, more studies should be conducted in a general population.

## Figures and Tables

**Table 1:** UTAUT final version obtained after Portuguese to English back-translation **.

Construct	Item Code	Item
Performance expectancy	PE1	Using the COVID-19 app will improve my knowledge about the hazard of being infected by COVID-19.
	PE2	I Consider the STAYAWAY COVID app would be helpful to evaluate the risks of contracting COVID-19.
	PE3	By using the STAYAWAY COVID app can the infection rate of COVID-19 be limited.
Effort expectancy	EE1	Learning to use the STAYWAY COVID app, will be easy for me.
	EE2	The STAYWAY COVID app, would not be difficult for me to use.
	EE3	I will rapidly be able to manage the STAYWAY COVID app. Using STAYWAY COVID app will be easy task for me.
Social influence	SI1	People that are important to me, think that I should use the STAYWAY COVID app.
	SI2	People that have influence over me, recommend that I use the STAYWAY COVID app.
	SI3	People whom I value their opinion, recommend that I use the STAYWAY COVID app.
Facilitating conditions	FC1	I have the necessary knowledge to use the STAYWAY COVID app.
	FC2	I have the necessary resources to use the STAYWAY COVID app.
	FC3	The STAYWAY COVID app is compatible with other apps on my smartphone.
Behavioral intention	BI1	I could be willing to use the STAYWAY COVID app.
	BI2	I plan on using the STAYWAY COVID app.
	BI3	I want to use the STAYWAY COVID app in the future
App-related privacy concerns	PC1 *	I am concerned over my privacy being exposed by the use of the STAYWAY COVID app.
	PC2 *	Using the STAYWAY COVID app will make me uncomfortable regarding any privacy exposure.
	PC3 *	I would be worried over my privacy being breached by using the STAYWAY COVID app.
Innovativeness	IN1	People seek my advice on new technology.
	IN2	Usually/In general, on my social circle I am the first to acquire new technology when it comes out.
	IN3	Usually, I can work out how to use new technology products without other’s help.
COVID-19 related stress	CS1	Even when I am busy with other things, I worry with the COVID-19 situation.
	CS2	The current COVID-19 situation/issue is very stressful for me.
	CS3	I am worried with the consequences of the crises provoked by COVID-19 pandemic.

* Items negatively ordered were reverse-scored for further analysis. ** Portuguese version (P-UTAUT) will be available upon request to authors.

**Table 2:** Socio-Demographic Characterization.

		Frequency *n* = 1081	(%)
Gender	Female	768	71.0
	Male	310	28.7
	Other	3	0.3
Age group	16–24	640	59.2
	25–34	155	14.3
	35–44	113	10.5
	45–54	96	8.9
	55–64	68	6.3
	More than 65	9	0.8
Educational qualifications	High School or Technological/Vocational Courses	381	35.2
	Bachelor’s degree	445	41.2
	Master’s degree	145	13.4
	PhD degree	97	9.0
	Other	13	1.2
Occupation	Student	845	78.2
	Teacher	164	15.2
	Other school staff	72	6.7

**Table 3:** Reliability and validity analysis of the P-UTAUT.

Construct	Item	Q1	Median	Q3	Factor Loadings (*λ*_ij_)	AVE	CR	Cronbach’s α
Behavioral intention (BI)	BI1	2	3	4	0.773	0.73	0.89	0.88
	BI2	1	2	3	0.852			
	BI3	1	2	3	0.936			
Performance expectancy (PE)	PE1	1	2	3	0.774	0.67	0.86	0.86
	PE2	2	3	3	0.874			
	PE3	2	2	3	0.800			
Effort expectancy (EE)	EE1	4	4	5	0.751	0.48	0.73	0.70
	EE2	4	4	5	0.773			
	EE3	3	4	5	0.516			
Social influence (SI)	SI1	1	2	3	0.867	0.72	0.89	0.88
	SI2	1	2	3	0.771			
	SI3	1	2	3	0.903			
Facilitating conditions (FC)	FC1	4	4	5	0.869	0.34	0.55	0.58
	FC2	3	4	5	0.368			
	FC3	3	4	5	0.337			
Innovativeness (IN)	IN1	2	3	4	0.497	0.36	0.60	0.62
	IN2	1	2	3	0.341			
	IN3	3	4	5	0.853			
App-related privacy concerns (PC)	PC1	2	3	4	0.967	0.87	0.95	0.95
	PC2	2	3	4	0.853			
	PC3	2	3	4	0.967			
COVID-19-related stress (CS)	CS1	3	4	4	0.740	0.45	0.69	0.68
	CS2	3	4	4	0.765			
	CS3	4	5	5	0.452			

Note: Q1 (Quartile 1), Q3 (Quartile 3), AVE (average variance extracted), and CR (composite reliability).

**Table 4:** Discriminant validity matrix: inter-construct correlations and the square root of AVE in the diagonal.

	PE	EE	SI	FC	PC	IN	CS	BI
PE	0.817							
EE	0.042	0.690						
SI	0.555 ***	0.028	0.849					
FC	−0.110 **	0.930 ***	−0.035	0.582				
PC	0.095 **	0.228 ***	0.059 ^†^	0.153 ***	0.930			
IN	−0.012	0.665 ***	0.045	0.652 ***	0.065 ^†^	0.603		
CS	0.208 ***	0.113 **	0.219 ***	0.035	0.005	0.032	0.668	
BI	0.753 ***	0.040	0.545 ***	−0.087 *	0.199 ***	−0.003	0.287 ***	0.856

Legend: Performance expectancy (PE); Effort expectancy (EE); Social influence (SI); Facilitating conditions (FC); App-related privacy concerns (PC); Innovativeness (IN); COVID-19-related stress (CS); and Behavioral intention (BI). Significance of correlations: ^†^ *p* < 0.100; * *p* < 0.050; ** *p* < 0.010; *** *p* < 0.001.

**Table 5:** Goodness of fit indices of P-UTAUT scale.

Fit Index	Recommended Value [14,19]	Initial Model	Modified Model
χ^2^	The lower its value, the better it is.	835.915	208.032
χ^2^/*df*	[2; 5]—Poor fit	3.732	2.600
CFI	≥0.95—Very good fit	0.955	0.988
TLI	[0.9; 0.95]—Good fit ≥0.95—Very good fit	0.944	0.984
RMSEA	≤0.05—Very good fit	0.050	0.038
SRMR	Values ≤ 0.08 are recommended	0.060	0.032

## Data Availability

The data presented in this study are available on request from the corresponding author. The data are not publicly available due to ethical restrictions.

## References

[1] OECD/European Union (2022). Health at a Glance: Europe 2022: State of Health in the EU Cycle.

[2] Walrave M., Waeterloos C., Ponnet K. (2020). Ready or Not for Contact Tracing? Investigating the Adoption Intention of COVID-19 Contact-Tracing Technology Using an Extended Unified Theory of Acceptance and Use of Technology Model. Cyberpsychol. Behav. Soc. Netw..

[3] Portugal In. Tech Innovation Hub. http://www.portugalin.gov.pt/innovation/.

[4] Instituto Nacional de Estatística (2020). Portal do INE—Proporção de Agregados Domésticos Privados com Pelo Menos um Indivíduo com Idade Entre 16 e 74 Anos e com Ligação à Internet em Casa. https://www.ine.pt/xportal/xmain?xpid=INE&xpgid=ine_indicadores&indOcorrCod=0001031&contexto=bd&selTab=tab2.

[5] Global Change Data Lab Our World in Data. https://ourworldindata.org/explorers/coronavirus-data-explorer.

[6] Kukuk L. (2020). Analyzing Adoption of COVID-19 Contact Tracing Apps Using UTAUT. Bachelor’s Thesis.

[7] Venkatesh V., Morris M.G., Davis G.B., Davis F.D. (2003). User Acceptance of Information Technology: Toward a Unified View. Manag. Inf. Syst. Q..

[8] Lu X., Zhang R., Zhu X. (2019). An empirical study on patients’ acceptance of physician-patient interaction in online Health Communities. Int. J. Environ. Res. Public Health.

[9] Naranjo-Zolotov M., Oliveira T., Casteleyn S. (2019). Citizens’ intention to use and recommend e-participation: Drawing upon UTAUT and citizen empowerment. Inf. Technol. People.

[10] LeRouge C.M., Hah H., Deckard G.J., Jiang H. (2020). Designing for the Co-use of consumer health technology in self-management of adolescent overweight and obesity: Mixed methods qualitative study. JMIR mHealth uHealth.

[11] Dar A., Lone A., Zahoor S., Khan A., Naaz R. (2020). Applicability of mobile contact tracing in fighting pandemic (COVID-19): Issues, challenges and solutions. Comput. Sci. Rev..

[12] Westcott R., Ronan K., Bambrick H., Taylor M. (2017). Expanding protection motivation theory: Investigating an application to animal owners and emergency responders in bushfire emergencies. BMC Psychol..

[13] Van de Vijver F., Hambleton R.K. (2006). Translating Tests: Some Practical Guidelines. Eur. Psychol..

[14] Sousa V.D., Rojjanasrirat W. (2011). Translation, adaptation and validation of instruments or scales for use in cross-cultural health care research: A clear and user-friendly guideline. J. Eval. Clin. Pract..

[15] Bolarinwa O.A. (2015). Principles and methods of validity and reliability testing of questionnaires used in social and health science researches. Niger. Postgrad. Med. J..

[16] Child D. (2006). The Essentials of Factor Analysis.

[17] Taber K.S. (2018). The Use of Cronbach’s Alpha When Developing and Reporting Research Instruments in Science Education. Res. Sci. Educ..

[18] Green S.B., Lissitz R.W., Mulaik S.A. (1977). Limitations of coefficient alpha as an index of test unidimensionality. Educ. Psychol. Meas..

[19] Niemand T., Mai R. (2018). Flexible cutoff values for fit indices in the evaluation of structural equation models. J. Acad. Mark. Sci..

[20] Saris W.E., Gallhofer I.N. (2014). Design, Evaluation, and Analysis of Questionnaires for Survey Research.

[21] Hooper D., Coughlan J., Mullen M.R. (2008). Structural Equation Modelling: Guidelines for Determining Model Fit. Electron. J. Bus. Res. Methods.

[22] Alavi M., Visentin D.C., Thapa D.K., Hunt G.E., Watson R., Cleary M. (2020). Chi-square for model fit in confirmatory factor analysis. J. Adv. Nurs..

[23] Hoque R., Sorwar G. (2017). Understanding factors influencing the adoption of mHealth by the elderly: An extension of the UTAUT model. Int. J. Med. Inform..

[24] Apolinário-Hagen J., Menzel M., Hennemann S., Salewski C. (2018). Acceptance of Mobile Health Apps for Disease Management Among People with Multiple Sclerosis: Web-Based Survey Study. JMIR Form. Res..

[25] Paganin G., Apolinário-Hagen J., Simbula S. (2022). Introducing mobile apps to promote the well-being of German and Italian university students. A cross-national application of the Technology Acceptance Model. Curr. Psychol..

[26] Rosi A., Vugt F.T., Lecce S., Ceccato I., Vallarino M., Rapisarda F., Vecchi T., Cavallini E. (2021). Risk Perception in a Real-World Situation (COVID-19): How It Changes from 18 to 87 Years Old. Front. Psychol..

[27] Franzen A., Wöhner F. (2021). Fatigue during the COVID-19 pandemic: Evidence of social distancing adherence from a panel study of young adults in Switzerland. PLoS ONE.

